# Optimization of lesser tuberosity osteotomy repair: a biomechanical assessment of suture tensioning, repair configuration, and type of suture

**DOI:** 10.1016/j.jseint.2025.08.001

**Published:** 2025-08-29

**Authors:** Mitchell H. Negus, Peter W. Kurtz, Megan Welsh, Shuchun Sun, Robert J. Reis, Brandon L. Rogalski, Richard J. Friedman, Jeremy L. Gilbert, Josef K. Eichinger

**Affiliations:** a Department of Orthopaedics and Physical Medicine, Medical University of South Carolina, Charleston, SC, USA; b Department of Bioengineering, Clemson University, Medical University of South Carolina Bioengineering Program, Charleston, SC, USA

**Keywords:** Total shoulder arthroplasty, Lesser tuberosity osteotomy, Suture, Mechanical study, Tensioning device

## Abstract

**Background:**

Lesser tuberosity osteotomy (LTO) is an effective method to manage the subscapularis for total shoulder arthroplasty but requires adequate compression and stability for healing after repair. The optimal configuration and type of sutures for LTO repair are unknown. The effect of variable surgeon strength on repair is similarly an unknown factor and may play a role in construct repair security. This study measured, in vitro, the effect of suture number and the effect of surgeon manual strength on repair. We hypothesized that increasing the number of sutures or selecting suture tapes over conventional sutures produces a stronger repair. Secondarily, we hypothesized that differences in surgeon manual strength introduce variability into the strength of the repair.

**Methods:**

A custom jig was used to mechanically test conventional #5 braided suture and 1.7 mm suture tape. Combinations of 1, 2, 3, 4, and 5 sutures of each type were preloaded, cyclically loaded, and loaded to ultimate failure. After measuring grip strength and traction strength of 3 orthopedic surgeons, we tested their hand-tied repairs and compared them to a self-tensioning suture cerclage system.

**Results:**

Increasing the number of sutures significantly increased the strength of repair in a relatively linear fashion (*P* < .001). Tests with #5 sutures showed a significant difference between 3 and 4 sutures (*P* = .005); however, those with suture tape did not. All 3 surgeons demonstrated significantly different grip strengths. Surgeon 2 showed greater traction strength than Surgeon 1 (*P* < .001) and Surgeon 3 (*P* < .001). Hand-tied repairs mirrored this trend, as those performed by Surgeon 2 were stronger than those by Surgeon 1 (*P* = .041) and Surgeon 3 (*P* = .028). A self-tensioning suture cerclage system was equivalent to all surgeons' hand-tied repairs.

**Conclusion:**

Four sutures appear to be an efficient number for LTO repair, as we observed no appreciable increase in strength with the addition of a fifth suture. There was no difference in strength between the suture types. Hand-tied repairs exhibit significant differences in strength among surgeons. If a surgeon considers this variation unfavorable, they may opt to use a tensioning device to ensure consistent repair strength according to their clinical judgment.

Total shoulder arthroplasty (TSA) is a surgical procedure that offers relief from several conditions, including arthritis^16^ and humerus fractures.^19^ Accessing the glenohumeral joint by detaching the subscapularis is a prominent component of the TSA surgical procedure. There are many different methods of subscapularis management available, including subscapularis tenotomy, subscapularis peel, lesser tuberosity osteotomy (LTO), and subscapularis sparing techniques.^24^^,^^26^ One such technique avoids subscapularis takedown by accessing the joint through the rotator interval.^1^ However, this method is technically challenging and not applicable to all patients.

Although the clinical outcomes of each of the repair techniques are similar, the rate of healing varies among the methods. LTO maintains the lowest risk of complication as a result of optimal exposure of the glenohumeral joint and ability to perform radiographic monitoring.^10^^,^^20^^,^^26^ Healing of the subscapularis is critical to the success of the surgery, as postoperative failure of the subscapularis results in failure of the TSA itself. A healed LTO is essential to a successful TSA outcome.^26^ An ideal repair technique is reproducible, cost-effective, simple, time efficient, and strong enough to allow immediate shoulder range of motion.

Existing literature generally supports the superior biomechanical strength of LTO compared to other subscapularis repair approaches.^13^^,^^18^^,^^21^^,^^22^ Although some studies have noted individual differences in LTO technique, few have directly compared them.^8^^,^^9^ For example, the optimal number of sutures, type of suture material, and method of suture tensioning/tying is unknown and likely has a direct effect on healing. Similarly, it is unknown whether techniques utilizing closed, self-tensionable suture tape constructs^7^ offer superior mechanical properties, while also requiring fewer holes in the proximal humerus than a traditional LTO repair. Many studies mechanically tested the subscapularis tendon (harvested from a cadaver) and determined metrics including initial, cyclic, and total displacement; ultimate strength; and stiffness.^11^^,^^23^

In this study, we assessed the resistance and consistency of suture strength for 1 to five sutures. This was done in duplicate using 2 different types of sutures. Then, we measured grip and traction strength of 3 orthopedic surgeons. Finally, we had each surgeon hand-tie an equal number of sutures and determined whether individual strength differences between surgeons affected the strength of repair. We asked (1) how does the number of sutures affect the tensile strength of a repair; (2) how does the type of suture affect the strength of repair; and (3) does a surgeon's strength affect the strength of a repair? We hypothesized that using more sutures for repair would increase strength, and suture tapes would provide greater strength of repair than conventional sutures. In addition, we hypothesized that repairs would vary in the maximum force they can withstand between surgeons with different levels of strength.

## Materials and methods

### Experimental setup

**Figure 1:**
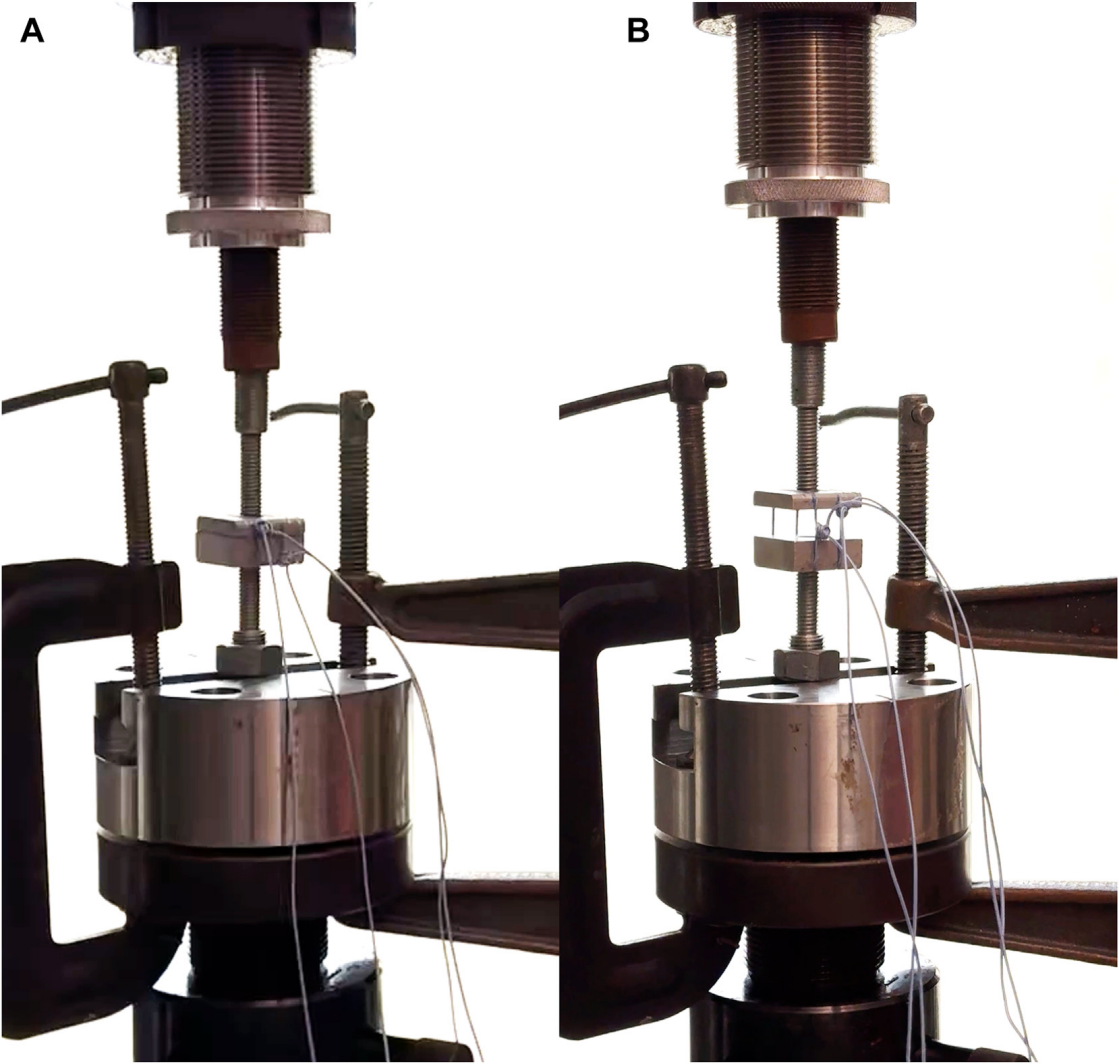
Mechanical testing setup. (**A**) Sutures secure both components of the jig together before testing begins. (**B**) Displacement of the sutures were aproximated by the displacement of the upper and lower jig components.

Testing was conducted using a 2-component LTO jig that was constructed from 2 grooved aluminum plates (Fig. 1*A*). The upper component represents the osteotomy fragment that is separated from the recipient humeral head (Fig. 1*B*).^15^ The five grooves in both components allow up to five sutures to be individually tied between the components. The jig was mounted on a materials testing system Bionix Servohydraulic benchtop system (Model 370.02; MTS Systems, Eden Prairie, MN, USA).

Biomechanical studies evaluating the strength of LTO repair use cadaveric specimens to replicate in vivo conditions.^2^^,^^4^^,^^9^^,^^11^, ^12^, ^13^^,^^18^^,^^20^^,^^23^^,^^24^ However, factors such as the creep of bony fragments under compression, the migration of sutures through bone and soft tissue, and other bone-suture interactions obscure the role that suture type, and the number of sutures, have on the strength of the repair. For these reasons, we chose an aluminum jig for the test apparatus. In addition, this model eliminates variability in bone morphology and osteotomy size to focus on suture characteristics, as osteotomy thickness may significantly alter biomechanical properties.^20^

### Mechanical testing

For the experiment, the sutures underwent a 10 N preload, followed by cyclic loading between 10 and 100 N at a rate of 9 Hz for a total of 500 cycles, as described in previous biomechanical studies.^7^^,^^14^ Immediately after cyclic loading, the sutures were loaded to failure at a rate of 1 mm/s^3^^,^^6^ Displacement (measured by the MTS) between the upper and lower component of the LTO jig was used to measure the displacement of the suture construct (Fig. 1*B*). During any phase of loading (eg during cyclic loading or load to failure, none of the sutures failed during the preload), a displacement of 1 mm from the starting position was considered the point of failure initiation. The force (N) required to reach 1 mm of displacement was recorded. During the load to failure, the force was recorded at 3 mm of displacement for each construct. Three mm of displacement was considered ultimate failure of the construct. The mechanism of failure was recorded at the end of each run. Force and displacement data were captured using MPE software (MTS TestSuite Multipurpose Elite software; MTS Systems, Eden Prairie, MN, USA). All mechanical testing took place in air at room temperature (21 **°**C).

### Suture testing

Five trials (n = 5) of mechanical tests were performed for each number of sutures (between 1 and 5) using both conventional #5 FiberWire (AR-7210; Arthrex, Inc., Naples, FL, USA) and 1.7 mm SutureTape sutures (AR-7511; Arthrex, Inc., Naples, FL, USA). The average cross-sectional area (n = 3) for each suture type was measured using digital optical microscopy. The cross-sectional area of the #5 FiberWire and 1.7 mm SutureTape was 0.63 ± 0.058 and 0.93 ± 0.11 mm^2^ respectively. The FiberWire sutures were selected to remain consistent with previous biomechanical studies using #5 sutures.^11^^,^^13^^,^^18^ We include 1.7 mm SutureTape because suture tapes may offer increased resistance to pull-through while maintaining comparable mechanical properties. Both suture types are routinely used by surgeons who perform LTO repairs.

After the sutures were tied using a surgeon's knot, a tensioning device (AR-1529 Suture tensioner with tensiometer; Arthrex, Inc., Naples, FL, USA) was used to apply a uniform tension of approximately 50 N to all five suture throws until the knot was completed. Force and displacement data were sampled at 40 Hz during mechanical testing and used to determine the maximum force the repair construct could withstand until failure. A displacement of 1 mm from the starting position was selected as the criterion for failure initiation. A displacement of 3 mm was considered the point of ultimate failure for the construct consistent with prior biomechanical studies.^9^

### Surgeon testing

**Figure 2:**
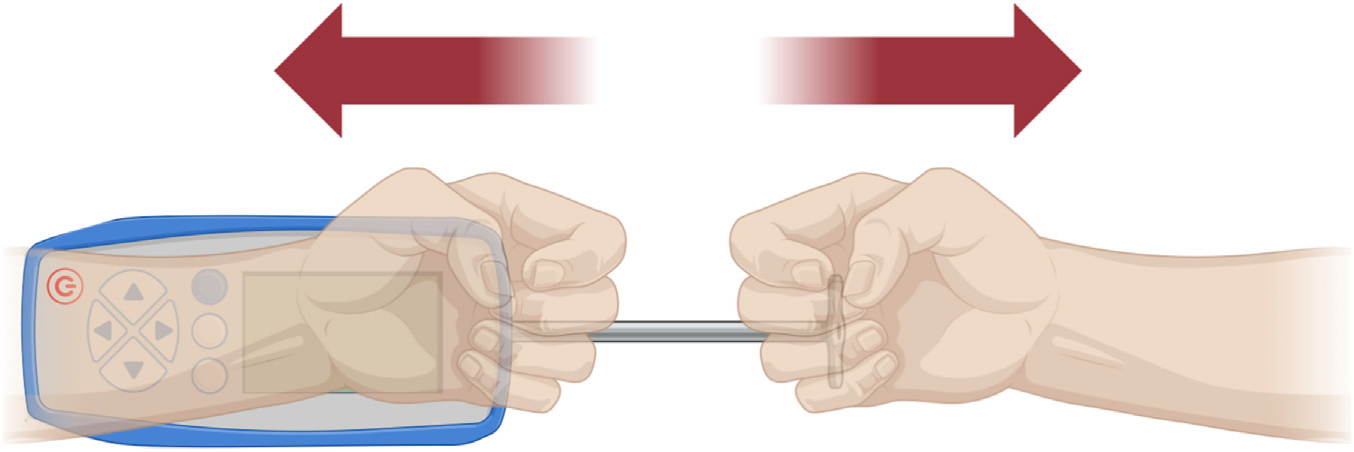
Traction test diagram. The surgeon holds the handheld digital dynamometer and grip attachment in each hand and pulls them apart at chest level.

We also examined the strength and variability of manual suture tying by comparing 3 different surgeons with differing grip strengths. All grip strength measurements were taken according to the AMA fifth Edition Guides to Impairment,^5^ with 3 (n = 3) grip strength measurements performed on each surgeon's dominant and nondominant hand. In addition, the surgeons performed a traction test on a digital axial dynamometer (n = 3). Holding the dynamometer in 1 hand and the grip attachment in the other at chest level, surgeons separated the 2, replicating the same motion used to hand-tie sutures in the operating room (Fig. 2). Following grip strength and traction strength tests, each surgeon completed five trials (n = 5) of mechanical testing with 4 hand-tied 1.7 mm suture tapes. The 3 surgeons' hand-tied sutures were also compared with the Arthrex FiberTape Tendon Compression Bridge (TCB), which is a self-tensioning suture cerclage kit implementing a handheld suture tensioning device (n = 5). Force and displacement data were collected and used to identify the maximum load before a displacement of 1 mm was reached.

### Statistical analysis

According to previous studies conducted on biomechanical testing of the subscapularis, accurately detecting differences in osteotomy displacement with adequate power (α = 0.05, β = 0.8) requires a sample size of at least 4 specimens.^25^ For the present study, we elected to complete five sets of strength tests per suture combination. Two-way analysis of variance with Tukey's multiple comparisons tests were performed to assess the effect of suture number and type on the maximum load for both 1 mm and 3 mm of displacement (α = 0.05). Simple linear regression was used to estimate the contribution of each additional suture to the strength of the repair construct after 1 mm of displacement. One-way analysis of variance with multiple comparisons was used to compare grip strength, traction strength, and repair strength between the 3 surgeons (α = 0.05).

## Results

### Suture testing

**Figure 3:**
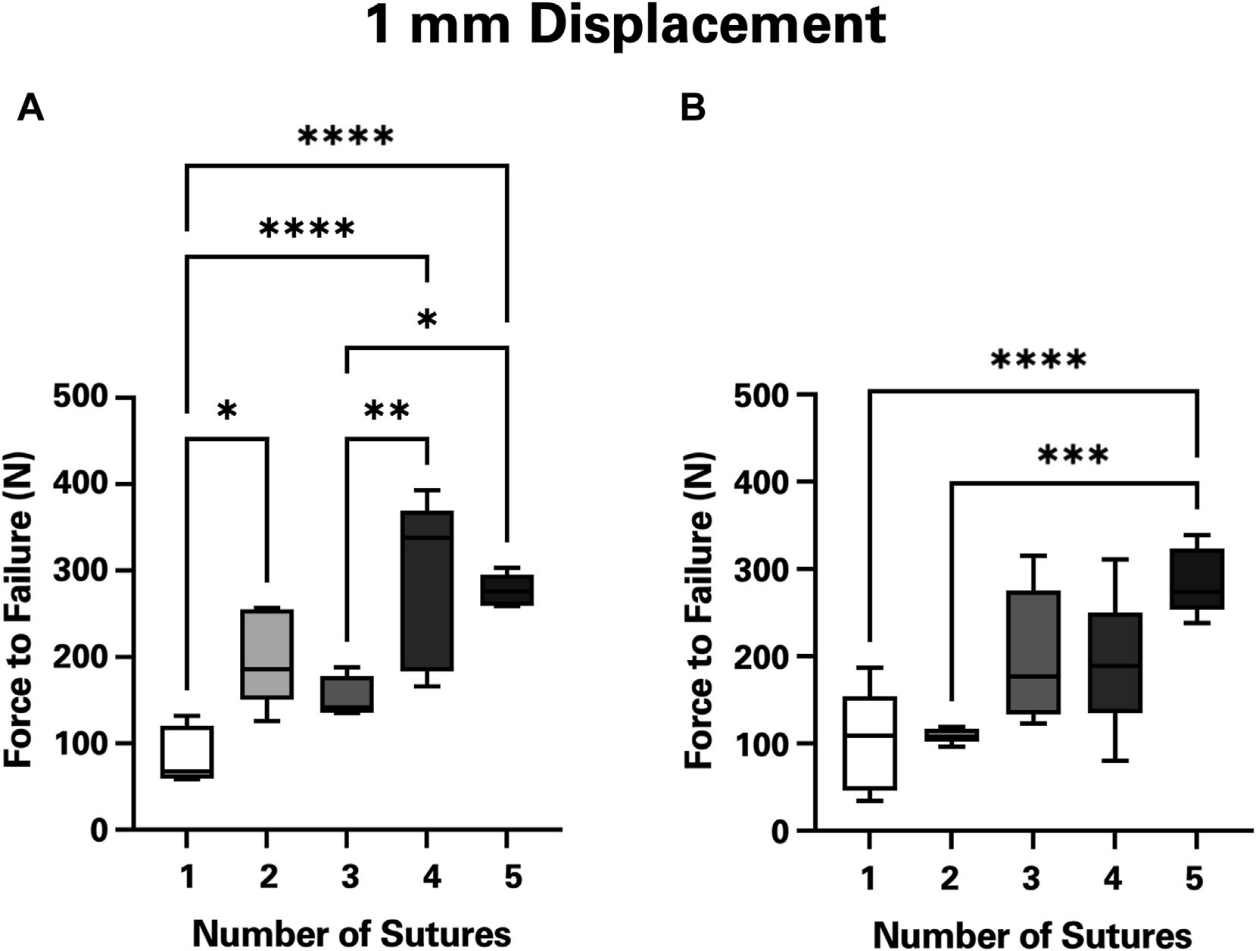
Mechanical strength for differing numbers of (**A**) conventional #5 FiberWire and (**B**) 1.7 mm suture tapes at the point of failure initiation (1 mm of displacement). As the number of sutures increased, so did the strength of the construct. This was true for both the #5 suture wire and the SutureTape. ∗ Indicates *P* ≤ .05. ∗∗ Indicates *P* ≤ .01. ∗∗∗ Indicates *P* ≤ .001. ∗∗∗∗ Indicates *P* ≤ .0001.

**Figure 4:**
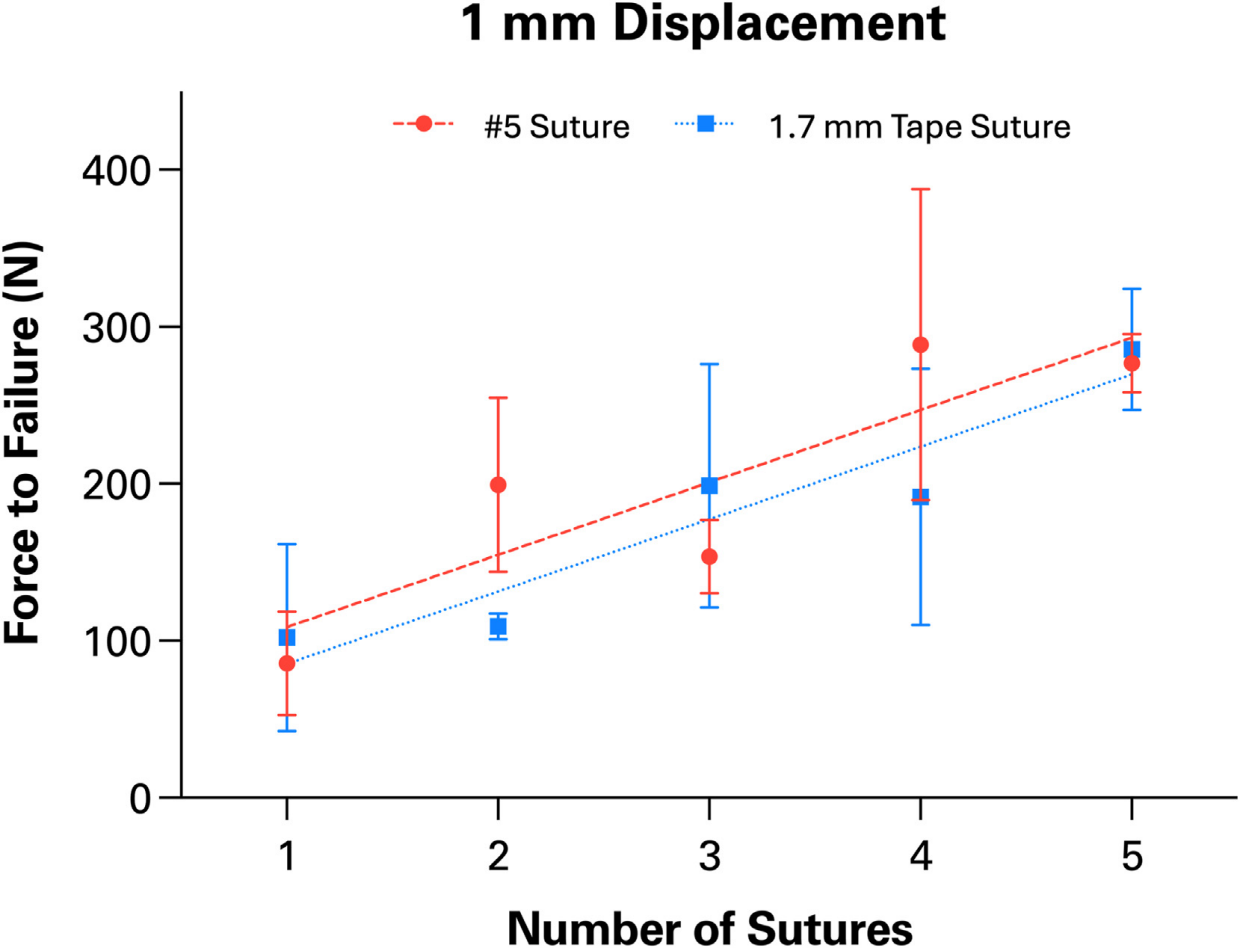
Linear association between the number of sutures and the strength of repair. With each additional suture added to the repair construct, strength may increase 47.2 N for traditional suture or 44.9 N for suture tape.

Increasing the number of sutures for both suture types significantly increased the strength of the repair after 1 mm of displacement. (*P* < .0001). Suture type alone had no impact on strength at the point of failure initiation (*P* = .16). However, certain configurations showed differences in strength arising from the interaction of suture type and number (*P* = .02). In these instances, #5 sutures were significantly stronger than suture tapes only in the 2 suture (*P* = .017) and 4 suture groups (*P* = .011). Constructs using #5 sutures significantly increased in strength from 3 to 4 sutures (*P* = .005) (Fig. 3*A*). When suture tapes were used, no differences were detected between repairs with 3 and 4 sutures (*P* > .99) (Fig. 3*B*). Additional comparisons between configurations using either suture type can be found in Table I. Regression analysis showed a linear association between the number of sutures and the strength of the repair (Fig. 4). Each conventional suture may allow the construct to withstand an added 47.2 N of force (*R*^2^ = 0.78). A single suture tape similarly contributes 44.9 N of strength to the repair (*R*^2^ = 0.89).

**Table I:** Mean strength values (Newtons) and statistical comparisons for each repair construct configuration at 1 mm of displacement.

Suture comparison	Conventional #5 braided suture			1.7 mm tape suture		
	Mean 1	Mean 2	*P* value	Mean 1	Mean 2	*P* value
1 vs. 2	85.5	199.4	.024∗	102.1	109.3	>.99
1 vs. 3	85.5	153.8	.34	102.1	198.8	.076
1 vs. 4	85.5	288.8	<.001∗	102.1	191.7	.12
1 vs. 5	85.5	277.0	<.001∗	102.1	285.6	.001∗
2 vs. 3	199.4	153.8	.72	109.3	198.8	.12
2 vs. 4	199.4	288.8	.12	109.3	191.7	.17
2 vs. 5	199.4	277.0	.22	109.3	285.6	.001∗
3 vs. 4	153.8	288.8	.005∗	198.8	191.7	<.99
3 vs. 5	153.8	277.0	.012∗	198.8	285.6	.14
4 vs. 5	288.8	277.0	>.99	191.7	285.6	.09

∗ Indicates statistical significance (*P* ≤ .05).

**Figure 5:**
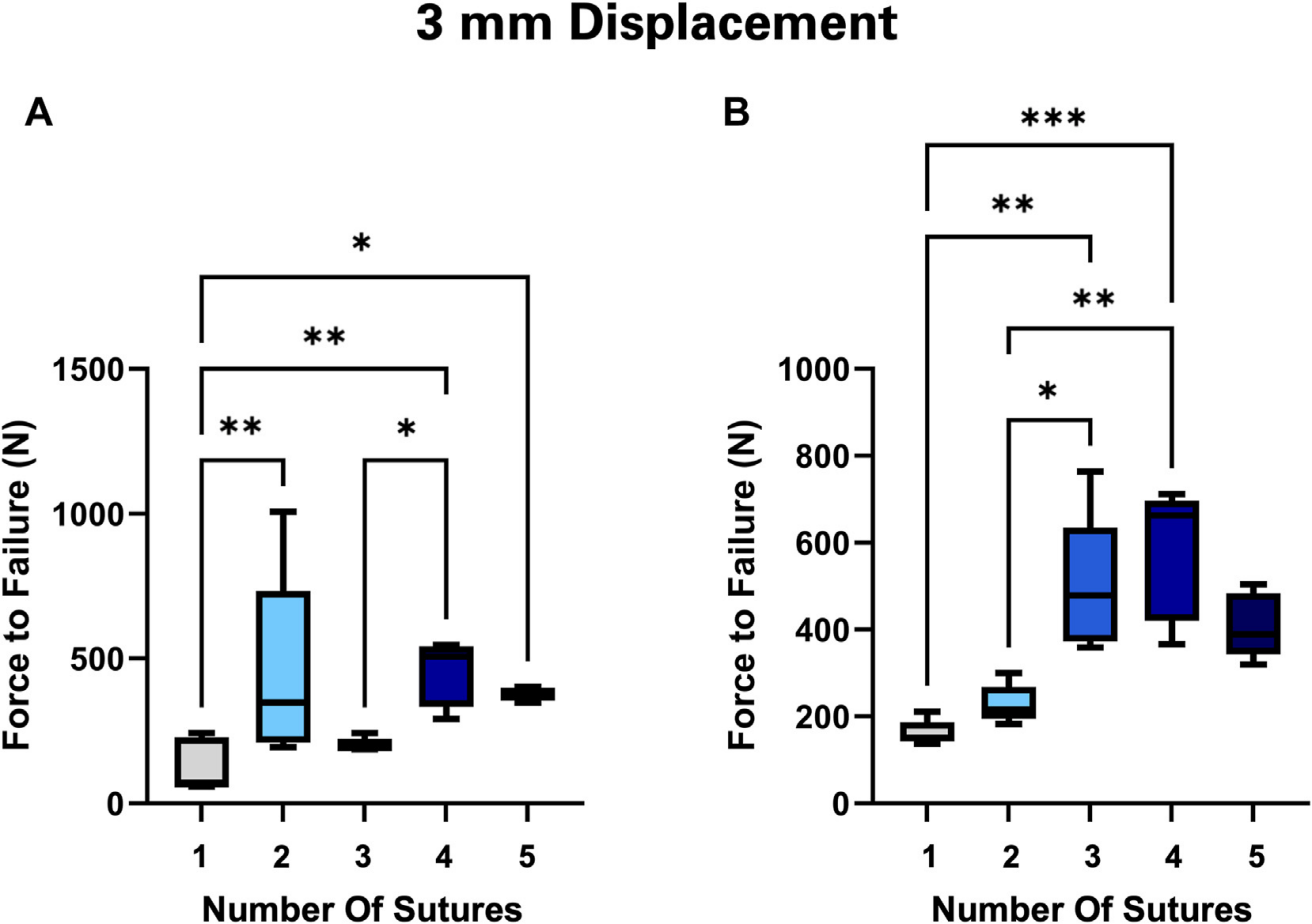
At the point of ultimate failure (3 mm displacement), both the #5 FiberWire (**A**) and SutureTape (**B**) had increased strength corresponding with the amount of sutures. This was a similar result to what was seen at the point of failure initiation (1 mm displacement, Fig. 3).

At the ultimate failure point of 3 mm of displacement, it was found that increasing the number of sutures correlated with an increase in construct strength (*P* < .0001). Like the previous results after 1 mm of displacement, there was no correlation between suture type and suture strength (*P* = .17). Again, certain configurations showed differences in strength arising from the interaction of suture type and number (*P* = .0038). A Tukey multiple comparison test revealed that the interaction occurred between the #5 wire and suture tape at 2 sutures and 3 sutures. The #5 wire was found to be stronger for the 2-suture construct while the suture tape was found to be stronger for the 3-suture construct (*P* = .016, *P* = .0015). For both the #5 wire (Fig. 5*A*) and suture tape (Fig. 5*B*), there was an increase in strength between constructs with 1 and 4 sutures (*P* = .0052, .0002). Further comparisons between groups can be seen in Table II. Sutures primarily failed by knot loosening combined with suture stretching.

**Table II:** Mean strength values (Newtons) and statistical comparisons for each repair construct configuration at 3 mm of displacement.

Suture comparison	Conventional #5 braided suture			1.7 mm tape suture		
	Mean 1	Mean 2	*P* value	Mean 1	Mean 2	*P* value
1 vs. 2	128.5	446.4	.006∗	162.6	228.3	.94
1 vs. 3	128.5	203.5	.91	162.6	498.7	.003∗
1 vs. 4	128.5	451.6	.005∗	162.6	579.7	<.001∗
1 vs. 5	128.5	378.1	.048∗	162.6	408.6	.053
2 vs. 3	446.4	203.5	.057	228.3	498.7	.027∗
2 vs. 4	446.4	451.6	>.99	228.3	579.7	.002∗
2 vs. 5	446.4	378.1	.93	228.3	408.6	.25
3 vs. 4	203.5	451.6	.05∗	498.7	579.7	.88
3 vs. 5	203.5	378.1	.28	498.7	408.6	.84
4 vs. 5	451.6	378.1	.91	579.7	408.6	.30

∗ Indicates statistical significance (*P* ≤ .05).

### Surgeon testing

**Figure 6:**
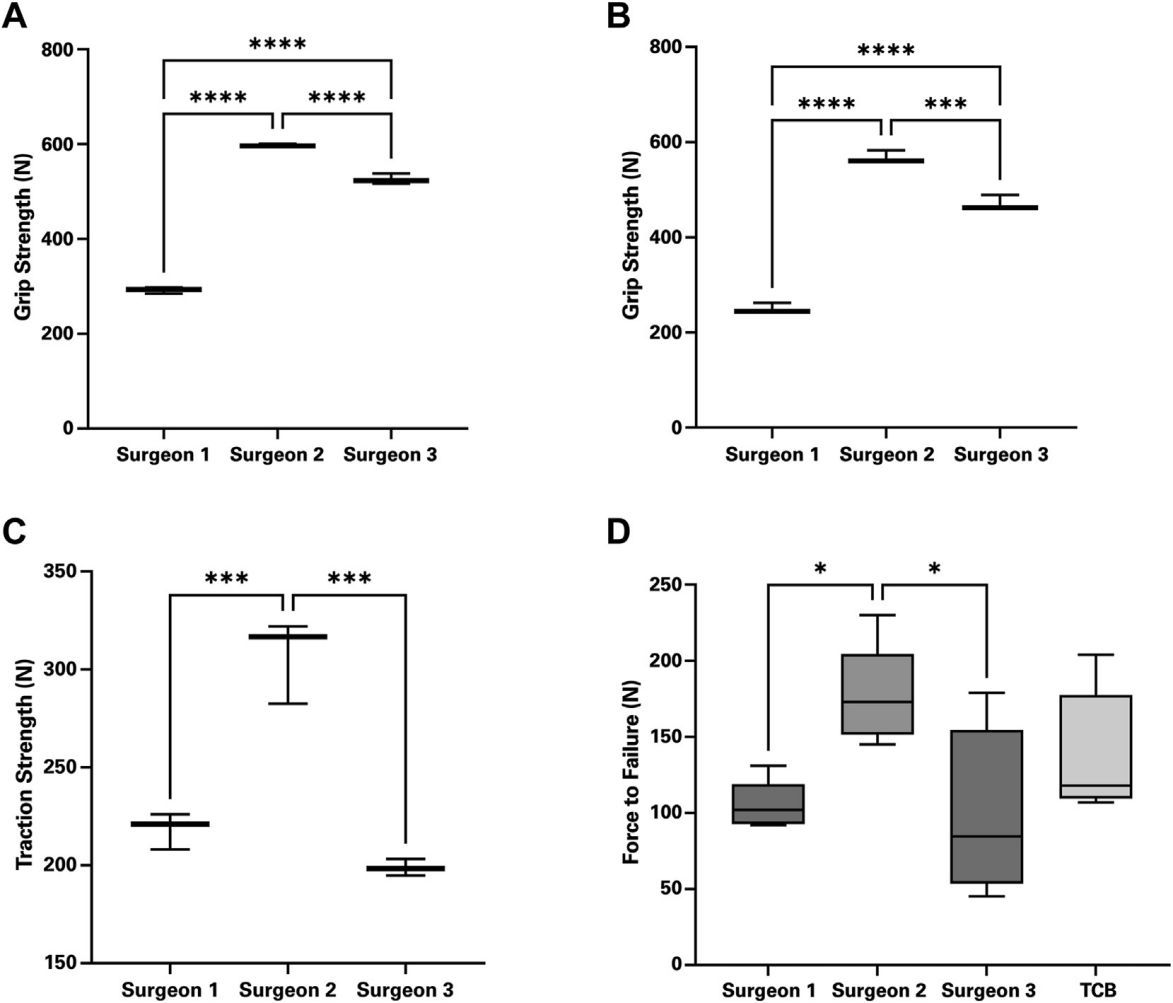
Individual surgeon metrics, including grip strength for each surgeon's (**A**) dominant hand, (**B**) nondominant hand, and (**C**) traction strength. (**D**) Strength of each surgeon's hand-tied repair compared to the self-tensioning tendon compression bridge. ∗ Indicates *P* ≤ .05. ∗∗ Indicates *P* ≤ .01. ∗∗∗ Indicates *P* ≤ .001. ∗∗∗∗ Indicates *P* ≤ .0001.

All surgeons showed statistically distinct grip strengths in their dominant and nondominant hands (Fig. 6 *A* and *B*). Surgeon 2 demonstrated significantly greater traction strength than both Surgeon 1 (*P* < .001) and Surgeon 3 (*P* < .001) (Fig. 6*C*). The test did not detect a significant difference in traction strength between Surgeon 1 and 3 (*P* = .26). Comparing the mechanical strength of the 3 surgeons' hand-tied sutures and the TCB after 1 mm of displacement, Surgeon 2 performed significantly stronger repairs than Surgeon 1 (*P* = .041) and Surgeon 3 (*P* = .028) (Fig. 6*D*). The TCB was found to be equivalent to each surgeon's hand-tied sutures. Failure only occurred by slackening of the sutures and subsequent displacement of the repair construct. All hand-tied sutures and TCB withstood cyclic loading prior to 1 mm of displacement.

## Discussion

One of the main findings of this study is that an increasing number of sutures increased repair construct resistance to failure, regardless of suture type (Fig. 3, Fig. 5). This was consistent after 1 mm and 3 mm of displacement.

At 1 mm of displacement, we observe a general increase in repair strength as sutures are added to the construct. The 2-suture and 4-suture FiberWire groups and the SutureTape 4-suture group show greater variation in strength. We propose that this variability arises because each construct's strength is limited by the weakest suture. When the weakest suture fails, the repair is dramatically weakened.

After 3 mm of displacement, the relationship between an increase in the number of sutures and construct strength was evident. Like the results after 1 mm of displacement, there is an imperfect comparison when looking at single suture increases. This is partially due to the high range seen in the #5 FiberWire 2-suture group and the SutureTape 3-suture group. Despite this, the data shows that as the number of sutures increases, so does the strength of the construct. This can be seen visually (Fig. 5) and is reinforced by statistical analysis (Table II).

The results of this study suggest a repair construct with at least 4 sutures is optimal. Mechanical testing with #5 sutures demonstrated that a 4-suture repair is significantly stronger than 1 with only 3 sutures after both 1 mm and 3 mm of displacement. In addition, for the #5 FiberWire and SutureTape sutures there was no statistical difference in strength between 4-suture and 5-suture repairs after both 1 mm and 3 mm of displacement. This finding suggests a 5-suture repair would not offer a discernible benefit over a 4-suture repair. Suture type did not affect strength of repair. This disproves our hypothesis that suture tapes offer greater repair strength than conventional sutures.

Existing biomechanical studies have used varying numbers of sutures for LTO repair.^17^ Repair constructs using 2 sutures,^2^^,^^11^^,^^18^ 4 sutures,^12^^,^^20^ and even 8 sutures^13^ have been evaluated in the literature. Our results indicate that LTO repairs with 4 sutures may provide greater strength than those with 2 sutures, particularly when SutureTape is used. These findings also raise the possibility that an 8-suture repair may not be necessary, as a 5-suture repair provides no apparent strength advantage compared to a 4-suture repair.

Suture displacement was approximated by the displacement between the upper and lower components of the aluminum LTO jig. While more sophisticated methods exist such as the use of an optical extensometer, with our methodology we were able to see a strong correlation between the number of sutures and construct strength at 1 mm and 3 mm of displacement for both #5 FiberWire and SutureTape (*P* < .0001, *P* < .0001). The displacement lengths of 1 mm and 3 mm were chosen in part not only because of the existing studies^9^ but also due to the lack of elastic elongation observed during testing. Observed 3 mm displacement of the jig corresponded with failure of the knot leading to slippage or permanent stretching to the suture. While values such as 3 mm of displacement are more synonymous with suture failure, we found that in most testing cases knot slippage or permanent stretching began at 1 mm. This is reinforced when looking at the similarities in data trends of both groups. We propose that the lack of observed elastic elongation is due to a combination of the metal jig, the consistent tension applied to each suture, and the loading speed of 1 mm/s during the load to failure.

A statistically significant variation in the strength of repairs between surgeons was identified after 1 mm of suture displacement. Repairs hand-tied by Surgeon 2 were able to withstand more force than those by Surgeons 1 and 3. The TCB kit, which uses a device to tighten the sutures, showed equivalent strength to each surgeon's hand-tied repairs with 4 suture tapes. This confirms the observations of Denard et al that a TCB approach is at least as effective as a standard 4-suture repair.^7^ All surgeons were found to have statistically distinct grip strengths in both their dominant and nondominant hands. These differences in grip strength did not align with the mechanical test results, as the repairs performed by Surgeons 1 and 3 were comparable in strength. Interestingly, the surgeons' pull-apart test results matched the strength of their repairs, as Surgeon 2 applied significantly more force to the apparatus than Surgeons 1 and 3. Tests of this type may be a useful way of assessing surgeon strength. To standardize the strength of surgical repairs, we recommend that surgeons should consider utilizing a device that assists with tensioning.

Although the presented model offers several insights into the effect of suture number on construct strength, it has some limitations. While this study was conducted within the context of LTO repair, and the sutures were tied by surgeons who perform this operation, we caution against overstating the findings of this work. The mechanical model used for this study was designed to magnify the effect of suture number. It does not replicate the physiological forces across an actual LTO repair and may not reflect true anatomic loading conditions. Indeed, by using a rigid aluminum jig, it is beyond the scope of this study to make any conclusions about how suture type or the number of sutures may influence suture-tissue and suture-bone interactions. To make more conclusions regarding in vivo performance, the test performed in this study would have to be repeated with a cadaveric or similar model. Despite this, the mechanical model reduces variability due to morphological size and bone quality differences with cadaveric models. In addition, the mechanical model allowed a large number of tests to be performed, which would not have been economically feasible with a cadaveric model.

Another limitation is that both components of the jig had 2 suture grooves on 1 side and 3 suture grooves on the other. Testing an odd number of sutures generated a slightly uneven force distribution over the repair construct, since the components are loaded at the center. This may have introduced variability into the measurements. The study demonstrated hand-tied surgical repairs vary in mechanical strength but did not directly assess the effects of using a tensioning device. Further studies may compare the strength of repairs when each surgeon ties sutures by hand and with the aid of a tensioning device.

Finally, the sutures, TCB, and tensioning device used in this study are products made by Arthrex, Inc., which provided funding for this work. Although this research was conducted independently from Arthrex, one of the authors received consulting fees from Arthrex for projects unrelated to this research. This relationship is disclosed in the interest of transparency and may present a potential source of bias.

## Conclusions

Greater numbers of sutures resist greater loads before failure, and a repair with at least 4 sutures may optimize strength when using conventional sutures or suture tapes. We did not observe differences in the strength of the repair between conventional #5 suture or suture tape. Repair constructs with hand-tied sutures vary significantly in strength and in relation to surgeon strength. Surgeons who believe that consistent tension is important may consider using a tensioning device during LTO repair procedures to minimize variation between surgeons.

## Disclaimers:

Funding: This work was supported by Arthrex, Inc. under grant #IIRR-018.

Conflicts of interest: Josef K. Eichinger has affiliations with organizations with direct or indirect financial interest in the subject matter discussed in the manuscript: consulting fees from Arthrex, Inc. Outside the submitted work, this he received personal fees from FH Ortho. Jeremy L. Gilbert, outside the submitted work, received consulting fees from DePuy Synthes, Smith and Nephew, and Naples Community Hospital. The other authors, their immediate families, and any research foundation with which they are affiliated have not received any financial payments or other benefits from any commercial entity related to the subject of this article.
